# Challenges and opportunities from using abortion harm reduction and value clarification and attitude transformation engagements for safe abortion advocacy in Uganda

**DOI:** 10.1186/s12978-023-01637-5

**Published:** 2023-06-28

**Authors:** Simon Peter Kayondo, Dan Kabonge Kaye, Stella Lovina Nabatanzi, Susan Nassuuna, Othiniel Musana, Imelda Namagembe, John Paul Nsanja, Jessica Morris, Hani Fawzi, Korrie de Koning, Jameen Kaur, Matthew Pretty

**Affiliations:** 1 Association of Obstetricians and Gynecologists of Uganda, P.O. Box 11966, Kampala, Uganda; 2 International Federation of Gynecologists and Obstetricians (FIGO), FIGO Headquarters, London, UK; 3 Royal Tropical Institute, Amsterdam, The Netherlands

**Keywords:** Unsafe abortion, Abortion safety, Abortion trajectories, Value clarification and attitude transformation, Advocacy for abortion care, Advocacy messaging, Advocacy strategies

## Abstract

**Background:**

From 2018, the International Federation of Gynecologists and Obstetricians (FIGO) implemented the Advocating Safe Abortion project to support national obstetrics and gynecology (Obs/gyn) societies from ten member countries to become leaders of Sexual and Reproductive Health and Rights (SRHR). We share experiences and lessons learnt about using value clarification and attitude transformation (VCAT) and abortion harm reduction (AHR) as strategies for our advocacy engagements.

**Methods:**

The advocacy goal of ending abortion-related deaths followed predefined pathways from an extensive needs assessment prior to the project. These pathways were strengthening capacity of the Obs/gyn society as safe abortion advocates; establishing a vibrant network of partners; transforming social and gender norms; raising awareness of the legal and policy environment regarding abortion, and promoting the generation and use abortion data for evidence-informed policy and practice. Our advocacy targeted multiple stakeholders including media, policy makers judicio-legal, political and religious leaders, health workers and the public.

**Results:**

During each engagement, facilitators required audiences to identify what roles they can play along the continuum of strategies that can reduce maternal death from abortion complications. The audiences acknowledged abortion complications as a major problem in Uganda. Among the root causes for the abortion context, audiences noted absence of an enabling environment for abortion care, which was characterized by low awareness about the abortion laws and policy, restricted abortion laws, cultural and religious beliefs, poor quality of abortion care services and abortion stigma.

**Conclusion:**

VCAT and AHR were critical in enabling us to develop appropriate messages for different stakeholders. Audiences were able to recognize the abortion context, distinguish between assumptions, myths and realities surrounding unwanted pregnancy and abortion; recognize imperative to address conflict between personal and professional values, and identify different roles and values which inform empathetic attitudes and behaviors that mitigate abortion harms. The five pathways of the theory of change reinforced each other. Using the AHR model, we delineate strategies and activities which stakeholders could use to end abortion deaths. VCAT enables critical reflection of views, beliefs and values versus professional obligations and responsibilities, and promotes active attitude and behavior change and commitment to end abortion-related deaths.

## Background

The World Health Organization defines unsafe abortion as a procedure for termination of a pregnancy done by an individual who does not have the necessary training (skills) or in an environment not conforming to minimal medical standards [1]. Despite scientific advances in provision of safe abortion at the primary care level, the persistently high unsafe abortion burden and resultant high burden of complications are a major cause of maternal death, morbidity and disability, as well as being a substantial cost to women, families, and health systems [2, 3]. While rates of unintended pregnancy have declined worldwide [4] with increasing access to and use of contraception likely contributing to these trend [5], some countries have persistently high rates. For instance, the estimated unintended pregnancy rates ranged from 11 (80% uncertainty interval: 9 to 13) in Montenegro to 145 (131 to 159) in Uganda per 1000 women aged 15–49 years [6]. Also, while the global unintended pregnancy rate declined between 1990 and 94 and 2015–19, the proportion of unintended pregnancies that end in abortion increased in countries where abortion is restricted, leading to stagnation in the global average abortion [5]. For instance, in countries that restricted abortion, the proportion of unintended pregnancies ending in abortion increased in every 5-year period, with a cumulative increase of 39% between 1990 and 94 and 2015–19, while the abortion rate increased by 82% [5].

An estimated 35 abortions occurred annually per 1000 women aged 15–44 years worldwide in the period 2010–14, which was 5 points less than 40 in 1990–94 [7]. Adjusting for population growth, the annual number of abortions worldwide increased by 5.9 million from 50.4 million in 1990–94 (48.6 to 59.9) to 56.3 million (52.4 to 70.0) in 2010–14 [7]. From 1990 to 2008, WHO used indirect estimation techniques to determine the incidence of abortion, both in countries with restrictive laws and in countries with more permissive laws in which many abortions occurred illegally and clandestinely [8], and all were labelled as unsafe abortions. Ganatra et al. [8] propose an approach in which they replace this dichotomous classification of abortion safety (unsafe versus safe abortion) with a three-tiered classification (safe, less safe, and least safe) which approach permits a nuanced description of the spectrum of varying situations that constitute unsafe abortion, and which also rhymes with the increasingly widespread replacement of dangerous, invasive methods with use of misoprostol outside formal health systems in legally restricted contexts [8]. Through using a model-based approach that caters for multiple factors that systematically affect abortion safety, this classification offers advantage of explicitly aligning both the operational estimation of safety categories with WHO’s conceptual definition of unsafe abortion and the technical standards of care outlined in WHO guidelines [8]. Of the estimated 55.7 million abortions that occurred worldwide each year between 2010 and 14, 30.6 million were safe, 17.1 million were less safe, and 8.0 million were least safe, making an estimated 25.1 million (45·1%), with 24.3 million (97%) of these in developing countries [8]. The proportion of less safe abortions was significantly higher in developing countries than developed countries (49.5% vs. 12.5%), and was also higher in countries with highly restrictive abortion laws than in those with less restrictive laws [8].

Pathways to abortion-related complications and effective care to prevent or mitigate them are complex in different contexts [9], necessitating a multipronged context-informed advocacy strategy that targets multiple predicting factors and stakeholders. Stakeholders’ understanding of the root causes and contextual factors related to unsafe abortion and challenges related to accessing safe abortion and abortion-related care is critical to ending abortion-related morbidity and mortality [9, 10]. Individuals’ personal experiences and relationships are interconnected within the broader context of their lives (such as gender, traditions and culture) [9]. Unmet need for family planning highlights a gap between women’s reproductive desire to avoid pregnancy and contraceptive behaviour [11]. Contraception is key to enabling couples and individuals exercise the right to decide freely and responsibly the number and spacing of their children. However, some women want to avoid unintended pregnancy yet do not use any contraceptive method, possibly because they do not have access to contraceptives or prefer not to use them for various reasons [11]. Deterring factors include ignorance about the methods, fear of side effects, gender-based violence, myths and misconceptions, high cost of methods [11]. Besides, even those using contraception may do so imperfectly, inconsistently or experience method failure. Other root causes for unintended pregnancy include low self-esteem, lack of autonomy, poverty and lack of women empowerment. Inequalities in accessing abortion-related care, are compounded by multiple individual characteristics including age, marital status, education level and inequitable gender relations [9, 12–14]. Indeed, women experience multiple, intersecting inequalities in both the risk factors for unwanted pregnancy (such as the negative traditional gender power relations and sociocultural factors) and the deficits in the healthcare system that hinder or delay access to abortion-related care [9, 13–18].

Abortion-related care-seeking can be understood as a process that responds to changing circumstances and experiences [9]. Patients’ delay to seek care, or healthcare providers’ delay to provide due quality timely abortion care (due to denial or refusal to provide women abortion-related counseling and services, partly due to abortion stigma), are some of the contributing factors to morbidity from abortion complications [9, 10, 17–19]. Advocacy that involves progressively changing national and international laws, policies, and patient management guidelines, coupled with the increasing decriminalization of abortion [10, 20–22] are some of the strategies in which access to safe abortion care may be promoted (to the highest standards of care and to the full extent allowable by law). Advocacy to provide data that informs strategies and or interventions is key to improvement in abortion policy and practice [23]. Additionally, targeted advocacy to provide access to the continuum of care from prevention of unintended pregnancy to management of abortion complications has potential to reduce abortion-related morbidity and mortality. To achieve this goal, there is need for advocacy targeting the different stakeholders who may influence behaviors that improve access to services or reduce abortion stigma, necessitating assessment of how advocacy targeting the different stakeholders may influence these behaviors. The objective of this paper is to share experiences and lessons learnt about using value clarification and attitude transformation (VCAT) and abortion harm reduction (AHR) as the advocacy theoretical frameworks for advocacy engagements in Uganda.

## The advocacy strategies

The abortion values clarification and attitude transformation (VCAT) is an intervention grounded in the Transtheoretical Model [24], which posits that an individuals’ beliefs influence their attitudes, and these attitudes then influence their motivations and intentions to enact specific behaviors in line with their held beliefs. Values play a critical role in determining how people make decisions and ultimately act [25]. Through VCAT, audiences engage in honest, open-minded and critical reflection and evaluation of personally-relevant abortion information and situations related to abortion practices or management, including the underlying factors which constitute the root causes of abortion.

Abortion harm reduction (AHR) is an evidence-based public health and human rights framework that prioritizes strategies to reduce harm and preserve health in situations where policies and practices prohibit, stigmatize and drive common human activities underground. The principles of AHR (neutrality, humanism and pragmatism) present a conceptual legal and ethical framework for comprehensive abortion care, where services can be provided for primary, secondary or tertiary prevention of abortion complications that cause death, through strategies and practices that address abortion related harms [26–28].

### Assumptions and strategies for advocacy engagements

The theory of change and advocacy strategies (indicated in Table 1) were identified through an extensive needs assessment process prior to the project [29] after which national societies developed their own society-specific action plans based on local contexts and priorities. These strategies were strengthening capacity of the Obs/gyn society as safe abortion advocates; establishing a vibrant network of partners; transforming social and gender norms; raising awareness of the legal and policy environment regarding abortion; and promoting the generation and use abortion data for evidence-informed policy and practice. The project design had to be adapted to conform with Uganda’s restrictive policy environment and AOGU’s diversity, with caveat on providing safe abortion services or actively advocating for reform of the legal and regulatory framework on abortion.

**Table 1:** Objectives, pathways for the theory of change and expected results

Objective	Pathway for change	Expected result
Strengthen AOGU’s capacity as safe abortion advocates; To strengthen AOGU as a professional organization that can drive and lead on change (pathway 1)	AOGU operationalized organizational policies and updated its strategic plan and priority statements in line with the advocacy project objectives	AOGU project staff (Project management unit) recruited and received FIGO-supported training on various aspects, including advocacy, communication, project management, monitoring and evaluation
Establish a coordinated and vibrant network of partners supportive of safe abortion (pathway 2)	To strengthen partnerships on advocacy	Strengthen coalition of like-minded partners as advocates to end morbidity and mortality from abortion
Transform the social and gender norms at all levels regarding safe abortion (pathway 3)	To create increased awareness about the root causes of abortion deaths, and to promote discussion of abortion through changing the narratives about abortion	Address the root causes of abortion deaths, including abortion stigma, through open discussion on abortion
Raise awareness of the legal and policy environment regarding abortion (pathway 4)	Awareness of the legal and policy framework will promote opportunities to identify critical roles to address the root causes of abortion-related deaths	Ability to promote abortion care and end abortion deaths to the full extent of available laws and policies
Promote the generation and use abortion data for evidence-informed policy and practice (pathway 5)	Available made available and widely disseminated to inform policy and practice	Information on abortion and opportunities for evidence-informed policies and practices

### The advocacy engagements

The advocacy targeted different stakeholders that included the media (print, broadcast, and social media bloggers), policy makers (in Ministries of Health, Justice, Education and Gender, Labor and social Welfare), judicio-legal department (police officers, prison officers and judges), politicians (parliamentarians and district local government officers), religious leaders, healthcare providers (district health officers and health workers working in both public and private healthcare institutions), traditional and cultural leaders, auxiliary healthcare providers (such as traditional birth attendants, traditional healers, village health teams, and herbalists), tutors from health professional institutions, members and leaders of civil society organizations, and the public.

## Methods

### Setting

From April 2019 to March 2022, the International Federation of Gynecology and Obstetrics (FIGO) worked with ten of its member associations (the national societies of obstetrics and gynaecology) to become key actors in safe abortion advocacy and national leaders in sexual and reproductive health and rights (SRHR) for women. The international Advocating Safe Abortion Project was implemented with national societies in ten countries: Benin, Cameroon, Côte D’Ivoire, Kenya, Mali, Mozambique, Panama, Peru, Uganda and Zambia. The Uganda project was nation-wide and was implemented in all regions of Uganda.

### Data collection during advocacy engagements

Each engagement involved introduction of the situation of abortion in Uganda, including the root causes of abortion, the legal, regulatory and policy environments for abortion care, individual and professional values that may impact on abortion care as well as the inherent value conflicts and how they can be addressed, the data needed to inform policy or practice, and the need for a concerted effort to end preventable morbidity and mortality from abortion complications. The sessions were held in the regions town where these persons resided, in a private venue hired for the function, and facilitated by a team of 3–4 facilitators. These groups were engaged separately, and in 5–6 engagements of about 30 persons per group, thus involving over 1500 people. At each session, the project team collected views on what commitments and strategies the audiences could propose to end abortion elated deaths, by considering the abortion trajectories and the continuum of care from prevention of unintended pregnancy though provision of safe abortion and management of abortion complications. Table 2 shows the process of value clarification for different stakeholder groups while Table 3 shows the commitments, made by the different stakeholders to end abortion related deaths along the possible continuum of care highlighted by the abortion harm reduction model. It highlights different commitments made by the different stakeholders, which elucidate on what behaviors may be adopted to end abortion-related deaths. The different stakeholders include, among others, the media, community mobilisers, parliamentarians and tutors from academic institutions.

**Table 2:** Anticipated value and attitude change for abortion care for suggested by the different stakeholders after VCAT, and the strategies for action in line of changed values

Audience	What they should think	What they should feel in relation to values	What they should do
Clients and patients	Need to appreciate the need for timely access to preventive and management services for abortion care	Should be aware of available services and what thy should expect and do in case of unwanted pregnancy or abortion complications	Access preventive measures of unwanted pregnancy Avoid risks of pregnancy termination Seek care for abortion complications
Lay public	There are root causes of abortion that need to be addressed There are gaps in service delivery Abortion stigma is rife	Urgent need to address root causes of abortion Need to improve deficits in abortion care service delivery Abortion stigma is rife	Advocate for or address root causes of abortion Advocate for improved abortion care within the legal and policy climate Address stigma
Tutors from academic institutions	Training curricula have gaps in content and competences that limit outcome-based training for service providers Training approaches are inadequate In-service training inadequate	Should identify a need to update training curricula to competence-based training Training approaches are inappropriate Trainees and providers lack competences	Should update training curricula to competence-based training Should improve training approaches Should assess competences
Policy makers in Health Ministry	Gaps exist in service delivery for abortion Data on abortion is deficient, incomplete or not timely Policies are not up-to-date	The importance of having accessible quality abortion services in place Data on abortion should be sought to inform policy and practice	Fulfil responsibility to ensure patients and communities can access relevant and timely services Collect and use timely abortion data for evidence-informed policies and practice
The Media	The importance of having the readers and viewers having information on abortion care that is not biased, accurate, empowering to clients representative and complete	Information on abortion is either not available, or if available, is biased, inaccurate, unrepresentative or incomplete	Ensure Information on abortion is available, and presented well, is unbiased, accurate, representative or complete Ensure abortion stigma is addressed
Politicians	Policies and laws should be available to ensure communities access information on abortion care, and clients can access equitable services	Currently, policies and laws not available to ensure communities access information on abortion care, and clients can access equitable services	Identify and lobby for Policies and laws to be available to ensure communities access information on abortion care, and clients can access equitable services
Healthcare providers	Need to appreciate the need for timely access to preventive and management services for abortion care, within the current legal and policy environment	Gaps in service delivery for timely access to preventive and management services for abortion care. Everyone needs a chance to be listened to, even if not supported	Their obligations and responsibilities to provide equitable, safe empowering services for the clients and patients, within the policy and legal framework
Religious leaders	Awareness of the root causes of abortion and their consequences including unplanned pregnancy, are often beyond control of the affected individuals Gaps exist in service delivery for primary, secondary and tertiary prevention of morbidity and mortality from abortion Abortion is highly stigmatized, limiting access to postabortion care	Religious leaders have a role to play in ending abortion morbidity and mortality There are root causes of abortion that can be prevented or mitigated. There is need for value and attitude change to end stigmatizing beliefs and values bout abortion	All stakeholders have a role address root causes of abortion All stakeholders have a role to play in advocating for service delivery that ensures access to primary, secondary and tertiary prevention of morbidity and mortality from abortion, within the legal and regulatory framework All stakeholders can address abortion stigma

**Table 3:** Commitments made by different stakeholders to address abortion related harms

Stakeholder	Commitment after advocacy
Tutors from health professional institutions	(a) To include detailed content of safe abortion and unsafe abortion context, VCAT and AHR in training curricula (b) To start providing this content to their trainees (c) Competence-based training on abortion care and family planning (d) Committed to include awareness of the legal, regulatory and policy framework on abortion in training curricula (e) Commitment to provide training on elective therapeutic abortions where indicated by laws and regulations
Media (print, broadcast, and social media bloggers)	(a) To start providing balanced and positive empathetic and non-judgmental stories about abortion to the public (b) To promote open discussion on abortion related issues
Policy makers in Ministries of Health	(a) To change the scope of practice for midlevel providers to include postabortion care (b) To update patient management guidelines to include abortion care (c) To provide abortion care within the full extent of the law and policies (d) To update the SRH policy guidelines (e) To include competence-based counseling training to providers
Policy makers from Ministry of Education and Gender, Labor and social Welfare	(a) To generate policies on adolescent SRH issues (b) To promote access to age-appropriate adolescent sexuality education (c) To promote strategies that address negative socio-cultural and gender factors that contribute to root causes of abortion (d) To promote strategies that address abortion stigma
Judicio-legal department (police officers, prison officers and judges)	(a) To support positive legal reforms related to abortion care (b) To support decriminalization of abortion care (c) To stop penalizing healthcare providers who offer postabortion care
Politicians (parliamentarians and district local government officers)	(a) To support positive law reforms related to abortion care (b) To support policies that address the root causes of abortion (c) To support all policies on provision of family planning and postabortion care (d) To promote strategies that address negative socio-cultural and gender factors that contribute to root causes of abortion (e) To promote strategies that address abortion stigma
Religious leaders	(a) To support provision of postabortion care (b) To support policies that emphasize primary prevention of abortion harms, especially religious guidance on behaviors, parenting and addressing negative socio-cultural practices (c) To reduce stigma on health-seeking behavior (d) For most groups, to support family planning including after abortion (postabortion family planning) (e) To support counselling at all levels, including for unwanted pregnancy clients to accept the situation and opt for antenatal care (f) To support strategies that provide rehabilitative counseling after abortion
Healthcare providers (district health officers and health workers working in both public and private healthcare institutions	(a) To provide non-judgmental postabortion care (b) To support contraception including postabortion (c) To provide non-judgmental counseling to all clients (d) To support efforts that address abortion stigma (e) To support policies that improve access to postabortion care irrespective of the circumstances of abortion (f) To provide abortion care within the legal and policy framework (g) To generate and use quality abortion data that informs practice and policy on abortion care (h) To participate in discussions that promote reduction of abortion stigma
Traditional and cultural leaders, traditional birth attendants and herbalists	(a) To support policies and strategies that address abortion stigma (b) To provide timely referral of abortion patients (c) To promote access to family planning including postabortion (d) To promote partnerships with the formal healthcare system
Members of civil society organizations	(a) To support open discussions on abortion (b) To address abortion stigma through community mobilization (c) To promote access to abortion care within the legal and policy framework (d) To promote policies that address the root cause of abortion (e) To promote access to SRH information for clients (f) To promote progressive decriminalization of abortion care
Village health teams	(a) To support policies and strategies that address abortion stigma (b) To promote access to counseling and antenatal care for women with unplanned or unwanted pregnancy (c) To provide timely referral of abortion patients for care or counseling (d) To promote access to family planning including postabortion (e) To promote linkages between with the formal healthcare systems, the communities and service providers withing the community
The public	(a) To promote open discussion about abortion issues (b) To promote access to family planning and postabortion care (c) To address or mitigate abortion stigma

### Post-advocacy engagement activities

After each engagement, the project team contacted the different stakeholders about 3 months after each engagement to assess how far they had gone in implementing their commitments, particularly. to identify what strategies they were able to implement. Additionally, through engagement with civil society and students from academic institutions, AOGU provided sub-grants for community development to specialized organizations, and small grants to the media and graduate students to promote evidence-based reporting on abortion and increase availability of abortion-related data respectively. The project team conducted midterm and end-term evaluation of the project, employing outcome harvesting as the evaluation method. The external evaluators used in-depth interviews among participants and stakeholders to assess the perceived impact the advocacy intervention. In case some positive (or negative) impact of the advocacy was suggested, the project team carried out outcome verification and substantiation. This involved confirmation of the advocacy outcome, assessing its significance, analysis of the project contribution, the actors, the change agents for this outcome and the likely impact on ending abortion-related deaths.

### Data analysis

Data was analyzed from project reports using textual analysis. Textual analysis refers to several research methods used to describe, interpret and understand texts including the assumptions, values revealed, symbolisms, and iteral meaning to the subtext [30]. Textual analysis is conducted to illuminate the underlying political, social or cultural context being investigated [30]. In order to understand individuals’ perceptions, you have to understand and analyze the way individuals attribute meaning to what goes on around them, or to find out how they react to action or lack of action [30]. Textual data analysis involved the following steps: analyzing spoken words (individuals’ spoken words turned into text), interrogating planned events (what are the motivations, predisposing factors and consequences of their action), identification of patterns and identifying the context of the statement or action, analyzing the variations or exceptions (and generating tentative explanations for the patterns and checking to see if they are present or absent in other settings or situations); working explanations into a theoretical model/framework (linking the commitments to a planned behavior).

## Results

In the VCAT role plays, participants were made to challenge deeply-held assumptions and myths; clarify and affirm their values and potentially resolve values conflicts; indicate how they would transform their beliefs and attitudes that impact behaviors; and state their intentions (as well as reasons why) to act in accordance with their affirmed values. Through this process, VCAT highlighted some of the root causes of stigma-related barriers to abortion service delivery safety, access and quality. Audiences whose attitudes shift from negative to neutral are less likely to obstruct and shall support or facilitate women’s access to care as well as promote the decriminalization of abortion. (Decriminalising abortion refers to the removal of specific criminal and/or civil sanctions against abortion from the law, so that no one is punished for having, providing or supporting access to abortion). Improvements in attitudes and behavioral intentions predict willingness to support or provide abortion care services, based on new values, improved understanding of women’s right to abortion and consequences of poor access to quality of care. From the evaluations conducted after each advocacy engagement, participants found them enlightening, educative, appropriate and suited to inform strategies to end abortion-related deaths. The specific strengths highlighted were inclusion of content on the context of abortion-related mortality, the law/regulations about safe abortion policies, safe abortion advocacy, the abortion harm reduction approach, and value clarification.

Tables 2 and 3 show the commitments made by the different stakeholders. Stakeholders readily identify roles to play according to their professional values and so were able to identify the personal values and how they conflict with professional values. As indicated by their commitments, stakeholders readily identified values related to primary and tertiary prevention of morbidity/mortality from abortion complications (rather than secondary prevention, which involves providing or promoting safe abortion). Through different VCAT role plays, specific audiences were able to recognize the induced abortion context, reflect and distinguish between myths and realities, recognize the imperative to address conflict between personal and professional values, and identify different roles audiences could play (as well the values that inform empathetic and positive attitudes) for lessening abortion harms.

Table 3 shows the anticipated attitude change and commitments after the VCAT sessions. The ultimate focus and goal of the advocacy was to reduce maternal mortality and morbidity from abortion complications. This message assisted in reducing opposition and helped to increase the understanding of the need for preventive measures targeting different levels according to the continuum of care depicted by the AHR model. Through VCAT, audiences were prompted to both identify the values that inform their beliefs and attitudes about abortion and contemplate about alternative values and their consequences. Initially, several participants expressed views blaming abortion patients, such as views that abortion “unacceptable”, is “evil” and a “sin”, and that “it follows misbehavior”, and “women wo conceive after rape should carry their cross”. These views changed after explaining the abortion context and trajectories, the root causes of abortion, the reasons why women die from abortion complications, and how death is preventable. While most participants expressed need for primary prevention of abortion-related harms (including support for contraception), secondary prevention (especially the option of antenatal care counseling for unintended pregnancy and tertiary prevention (through ensuring access to postabortion care services), some participants, mainly political and religious leaders, held negative beliefs and views about promoting abortion, which contrasted with their overwhelming commitment to promote addressing root causes of abortion or mitigation of abortion stigma.

During the midterm and end evaluation participants showed appreciation of evidence provided on how abortion significantly increased women and girls dying, clarification of the abortion law/regulation and explanation of how abortion stigma and health workers behaviour contributed to the deaths from abortion. Most respondents interviewed during the evaluation testified to changes in attitudes after the advocacy engagements, and reported changes in their behaviour. This included empathetic attitudes towards women with unintended pregnancy, greater acceptance of the need for safe abortion care, not being abusive towards women accessing care for post abortion complications, referral for safe abortion, and acceptance of community dialogues about supporting girls and women during pregnancy and after post abortion care.

There were several missed opportunities and challenges. Firstly, there was limited political will from the parliamentarians and district political leaders for legal reforms, though decriminalization of abortion was deemed acceptable to different stakeholders. However, the exact meaning of decriminalization of abortion—and what this entailed—varied for different stakeholders. Besides, the fact that the project was not directly advocating for change in the abortion law, nor directly providing safe abortion services, occasionally put AOGU at crossroads with partners that were actively advocating for these recommendations. Secondly, there was identified need to skill health service providers in provision of abortion care services countrywide in addition to training them in advocacy. There was identified need to support health facilities and providers with commodities for safe abortion. Besides, disagreement with the advocacy strategy and implementation was rife including from some AOGU members. Also, some implementing partners and stakeholders had different views about commitments, targets and timelines about advocacy, and often their views and values diverged from our advocacy strategy.

## Discussion

Our advocacy shows that with the appropriate advocacy message frame, different stakeholders will commit to becoming advocates for ending unsafe abortion-related mortality and morbidity, even in contexts where opposition is anticipated beforehand. Framing advocacy messages is key to successful advocacy. The AHR and VCAT delineate different roles stakeholders can play according to their values and beliefs as they elucidate abortion trajectories. The project was able to realize some achievements, such as stakeholder commitments to support changes for ending morbidity and mortality from abortion complications. Secondly, the project mobilized many AOGU members and stakeholders to accept the need to proactively implement activities at different levels to end the preventable morbidity and mortality from abortion complications. Thirdly, the project reaffirmed the need to design or reframe advocacy messages for different stakeholders. Advocates should not inflexibly stick to one message frame but should employ a frame that resonates with values of the message recipients. The engagement of multiple stakeholders enabled us to test run the messages so as to get different perspectives of diverse stakeholders. Whereas some messages are deemed relevant, important and touching by all stakeholders, others don’t move or touch them at all. A tailored approach is required for engaging different stakeholders—depending on what is perceived as the stakeholder’s interests, attitudes, beliefs and values—and identification of that entry point of interest for each stakeholder was key to advocacy. The project influenced a learning environment within the society. From participants’ evaluation of each engagement, the s lessons learnt from one meeting/VCAT were used to inform the he planning and conduct of subsequent engagements.

The findings affirm the view that while healthcare providers and other stakeholders are gate keepers to the full range of SRH services, they can, through VCAT and AHR counseling, make commitments that increase uptake of different SRH services (that could potentially reduce abortion-related deaths) by ensuring accessibility, availability, affordability and quality of the services. Secondly, VCAT training embedded within training on abortion harm reduction may improve positive values and nurture empathetic attitudes. For instance, values clarification workshop conducted among residents at Catholic training programs resulted in more residents endorsing more empathetic and positive attitudes toward abortion patient scenarios, even when training is insufficient. especially within deeply religious institutions and contexts [31]. Thirdly, the AHR elucidates on available options for clients with unintended pregnancy which include acceptance of the pregnancy and continuation with antenatal care or safe abortion. Indeed, prior intention to terminate a pregnancy does not compromise healthcare (and may even promote uptake of antenatal services) [32], when healthcare providers provide empathetic counseling. Besides, antepartum contraceptive counseling increases uptake of postpartum contraception [33]. Lastly, abortion advocacy needs to employ multifaceted strategies targeting multiple stakeholders with apropriate narratives [34–36]. The approach used in this advocacy project to adjust terminology, reframe advocacy messaging, tailor advocacy messages to the different stakeholder values is critical to illuminating what strategies and activities different stakeholders may employ to reduce abortion-related harms.

The AHR model highlights opportunities that make abortion available and feasible through selfcare [16, 37, 38]. While providing access to safe abortion is critical [39, 40], providing evidence and enhancing understanding of root causes, mechanisms, triggers and consequences of abortion-related morbidity from the perspective of different stakeholders may contribute to an enabling environment for management of abortion and other SRH services. In this enabled environment, the root causes of unintended pregnancy (which apparently intersect with the trajectories of abortion-related deaths) and consequences of unsafe abortion are also addressed. Regulation of the abortion care may improve access to safe abortion. Even in restrictive contexts, healthcare provider and other stakeholders’ awareness of the abortion laws and regulations increases opportunities to maximize access to safe abortion, as well as elucidating on situations in which abortion may be decriminalized. While the fact that the advocacy was not focused on changing the abortion law or directly providing safe abortion services led to some conflict with partners that were actively advocating for these recommendations, the more inclusive position related to the strategies made joint activities with some stakeholders more acceptable. Nevertheless, raising stakeholder awareness of legal and policy environments was an indirect call for the need to regulate abortion care.

As a limitation of our findings, the advocacy engagements stimulated stakeholders to identify and make commitments to end abortion-related deaths. The results of the advocacy engagements and ensuing commitments might not necessarily translate into actions that will lead to realization of the advocacy goal of ending abortion-related deaths. We cannot assume nor do we have any basis from the outcome harvesting, that the commitments made during the engagements would be fully implemented to enable increased access to safe abortion or reduction in abortion-related deaths. However, the commitments are a critical initial step, and the inclusiveness of the commitments show that, through value clarification, different stakeholders can identify a role to play along the abortion trajectory and the continuum of abortion care specifically and sexual and reproductive health services in general, along the AHR model.

## Scaling up assessment for the abortion harm reduction and VCAT intervention

Scaling up an intervention refers to expanding or replicating pilot or small-scale projects to reach more people and/or broaden the effectiveness of an intervention to a national level or to even more countries. Scaling up refers to *“deliberate efforts to increase the impact of successfully tested health interventions so as to benefit more people and to foster policy and program development on a lasting basis”* [41]. In the context of our findings, scale up refers to expanding advocacy among different stakeholders to seek commitments to increase access to SRH services (along the abortion trajectory) and eventually reduce abortion-related deaths, via strategies that include increased access to safe abortion. The scalability of an intervention is influenced by its effectiveness, the likely reach and adoption of the intervention, the likely costs of implementing at a larger scale, the acceptability and fit of the intervention within the local policy context [42]. Scaling up recognizes that different pathways may be used to achieve replication, adoption, adaptation or reach of an intervention [43].

Our abortion harm reduction and value clarification and attitude transformation intervention are credible in that they are derived from public health strategies of primary, secondary and tertiary prevention of illness (which is our context is prevention of mortality from abortion-related complications). The intervention is inclusive, and identifies roles for different players among the key stakeholders to provide the continuum of care, while addressing the factors in the abortion trajectory from the root causes of unintended pregnancy to management of abortion complications and linkage to other reproductive health services. Increasing access to safe abortion is a key component of the strategies. Besides, through VCAT, different stakeholders are encouraged to adapt empathetic values and attitudes, and address professional and personal vale conflicts, so as to provide care/services that provide the continuum of comprehensive abortion care. The support for change is likely to be high. The scaleup model has advantage over existing practices in that it expands options for increased access to safe abortion and addresses both the contributory factors for unintended pregnancy and the abortion complications that lead to abortion mortality and morbidity. Additionally, through advocacy, the intervention can be adapted to different abortion care contexts, irrespective of the existing legal and regulatory framework.

The framework for scaleup of the harm reduction intervention recommended is the one suggested by Barker et al. [44] which describes three core components: *“a sequence of activities that are required to get a program of work to full scale, the mechanisms that are required to facilitate the adoption of interventions, and the underlying factors and support systems required for successful scale-up”.* The four steps in the sequence include the first step: *Set-up*, which prepares the ground for introduction and testing of the intervention that will be taken to full scale. Our findings have partly covered this step. In the context of our intervention, the step involves sustained advocacy about the need to reduce abortion-related morbidity and mortality, through changing the abortion narrative from abortion being a medical problem to being a public health problem, where different players can identify definite roles along the abortion trajectory. The second step, D*eveloping the Scalable Unit*, an early testing phase, where results of the intervention are disseminated to seek opinions and recommendations (from diverse stakeholders) that inform scale up at a bigger level. The third step, *Test of Scale-up*, will test the intervention in a variety of settings and among the diverse stakeholders who potentially have a role along the abortion trajectory (from unintended pregnancy to death from abortion complications) and who represent different contexts that will be encountered in practice. The last step, *Go to Full Scale*, will enable replication or adaptation of the intervention among different stakeholders and different settings/contexts.

## Conclusions

In addition to liberalizing abortion laws and providing safe abortion services, targeted advocacy efforts can contribute to reduction of abortion-related morbidity and mortality if they generate commitments for implementation of interventions that address the full spectrum of abortion continuum from unintended pregnancy to management of abortion complications. This project demonstrates how VCAT and AHR with different kinds of stakeholders can help stakeholders understand the abortion context including the need to change to positive values that may lessen abortion-related harms. The findings elucidate how advocacy strategies may affect different stakeholder behaviors, and what changes might be needed to increase advocacy effectiveness. The five pathways of the theory of change for advocacy reinforced each other in elucidating on women’s abortion trajectories for the different stakeholders, and enabled framing of appropriate advocacy messages and commitments for strategies to end morbidity and mortality from abortion complications. The AHR and VCAT enabled critical reflection of individual views, beliefs and values versus professional obligations and responsibilities, and promoted active attitude change towards ending the spectrum of risk and vulnerability factors that shape, modify or predict abortion harms that contribute to the deaths from abortion complications. While there is compelling need to extend the coverage of training for comprehensive abortion care, there is need to include training in VCAT and AHR for all the different stakeholders. Secondly, there is need to build synergy among the different stakeholders, the goal of whose efforts should be prevention, mitigation or ending the preventable deaths from abortion complications. Using VCAT and AHR, all stakeholders can identify a role to play along the multifaceted strategies to end death from abortion complications.

## Data Availability

Not applicable.

## Abbreviations

AHR: Abortion harm reduction
AOGU: Association of Obstetricians and Gynecologists of Uganda
APMM: Advocacy for prevention of morbidity and mortality
CAC: Comprehensive abortion care
FIGO: International Federation of Gynecologists and Obstetricians
KIT: (The) Netherlands Royal Institute
PAC: Postabortion care
SRH: Sexual and Reproductive Health
SRHR: Sexual and Reproductive Health and Rights
VCAT: Value clarification and attitude transformation
